# Global disparities in the regulation of electronic cigarettes

**DOI:** 10.18332/tid/211968

**Published:** 2025-12-10

**Authors:** Catherine O. Egbe, Sharon Nyatsanza, Omotayo F. Fagbule

**Affiliations:** 1 Mental Health, Alcohol, Substance Use and Tobacco Research Unit, South African Medical Research Council, Pretoria, South Africa; 2 Department of Public Health, Sefako Makgatho Health Sciences University, Pretoria, South Africa; 3 School of Nursing and Public Health, University of KwaZulu-Natal, Durban, South Africa; 4 National Council Against Smoking, Pretoria, South Africa; 5 Department of Periodontology and Community Dentistry, Faculty of Dentistry, College of Medicine, University of Ibadan, Ibadan, Nigeria; 6 School of Public Health, University of Nevada Reno, Reno, United States

**Keywords:** electronic cigarettes, legislation, tobacco control policy, global policy, regulations

**Dear Editor,**

Authorities facing aggressive e-cigarette marketing to vulnerable populations^1,2^ often fall back on legislations predating e-cigarettes^3^ to regulate these products. We investigated disparities in e-cigarette regulations/bans for countries in the six WHO regions and four World Bank income groups.

Countries’ regulations/bans on e-cigarettes were coded Yes (1) or No (0) for presence of a regulation/ban using the Institute of Global Tobacco Control, Johns Hopkins Bloomberg School of Public Health^4^, and Global Center for Good Governance in Tobacco Control websites^5^. For countries listed as ‘unknown’ on both websites, we checked government and other online sources for e-cigarette regulations. Countries’ WHO region^6^ and World Bank income group categories^7^ were entered in a spreadsheet. Frequency distributions and chi-squared tests were conducted using SPSS v26. Independent bivariate logistic regression analyses used the WHO region and World Bank income group as predictor variables with significance at p<0.05.

Among 194 ‘countries’ (193 WHO member states/entities and Niue), 66.5% have e-cigarette regulations/bans (Figure 1). All 53 countries in the WHO Europe region have regulations, 15/47 in the AFRO region, 8/11 in South-East Asia, 23/35 in the Americas, 18/27 in the Western Pacific, and 12/21 in the Eastern Mediterranean. Using the World Bank income grouping, 54/60 high income countries have regulations, 39/53 upper middle-income countries, but only 6/26 low-income countries (LICs), and 29/54 lower middle-income countries, have e-cigarette regulations/bans (Figure 1). Chi-squared test showed a significant association between having an e-cigarette regulation/ban and belonging to a WHO region/World Bank income group (p<0.001).

**Figure 1:**
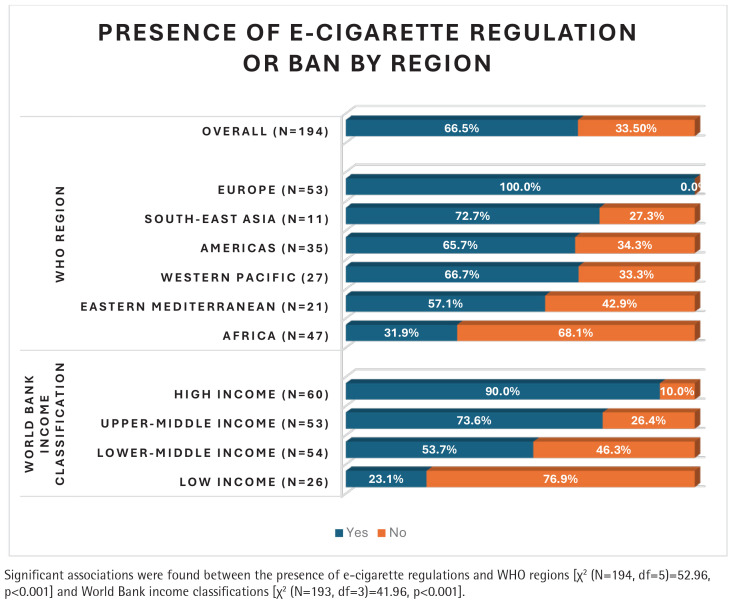
Proportion of countries with or without electronic cigarette regulation or ban, categorized by WHO region and World Bank income classification

Bivariate logistic regression showed countries outside Africa had significantly higher odds of having e-cigarette regulations: nearly six times in South-East Asia, four times in the Americas and Western Pacific, and three times (not significant) in the Eastern Mediterranean. WHO Europe was excluded, as all its countries have regulations. Further, compared to LICs, HICs had 30 times, upper middle-income nine times, and lower middle-income nearly four times higher odds of having e-cigarette legislation.

Since the market debut of e-cigarettes in 2004^8^, many LICs and lower middle-income countries do not yet have regulations. Lagging tobacco-control laws/regulations allow normalization of these products through unrestricted advertising, and targeted marketing.

Africa and LMICs (often overlapping WHO and World Bank categories) disproportionately lack e-cigarette regulation – reasons may include less political and economic stability, low public awareness, and high tobacco industry interference^9^. Some LMICs lack the political will and finances to oppose industry interference, but may benefit from regional cooperation.

All WHO Europe region countries have some form of e-cigarette regulation. The European Union (EU) regional bloc promotes vertical policy diffusion within countries, e.g. the Tobacco Products Directive (2014/40/EU) governs EU sales of e-cigarette as consumer products^10^, shaping national policies fostering harmonization and monitoring of tobacco and e-cigarette regulations across the EU^10^. The EU example shows how regional blocs like the African Union, and the Association of Southeast Asian Nations (ASEAN) can work together cost-effectively to strengthen political will for stronger laws.

Regulatory frameworks, while crucial, may not always address underlying inequities effectively, therefore robust surveillance and effective policy implementation are needed.

## Supplementary Material



## Data Availability

The data supporting this research can be found in the Supplementary file.
