# Supplementary material for: Global disparities in the regulation of electronic cigarettes
Source: Tob Induc Dis. 2025 Dec 10;23:10.18332/tid/211968. doi: 10.18332/tid/211968 (PMC12687329; doi:10.18332/tid/211968)
Supplement: Supplementary file 1 [file TID-23-191-s1.pdf]

Supplementary Table (S1): E-cigarette regulatory status by country

Entity	WHO region	World Bank Income group	Institute of Global Tobacco Control Website*	GGTC E-CIGARETTE BAN & REGULATION: Global Status as of Oct. 2023**	Other sources$^{\beta}$	Final decision on Legislation ***
Afghanistan	Eastern Mediterranean	Low income	Unknown			0
Albania	Europe	Upper middle income	Yes	Yes		1
Algeria	Africa	Lower middle income	Yes	Yes		1
Andorra	Europe	High income	Yes	Yes		1
Angola	Africa	Lower middle income	Unknown			0
Antigua and Barbuda	Americas	High income	Unknown			0
Argentina	Americas	Upper middle income	Yes	Yes		1
Armenia	Europe	Upper middle income	Yes	Yes		1
Australia	Western Pacific	High income	Yes	Yes		1
Austria	Europe	High income	Yes	Yes		1
Azerbaijan	Europe	Upper middle income	Yes	Yes		1
Bahamas, The	Americas	High income	Unknown			0
Bahrain	Eastern Mediterranean	High income	Yes	Yes		1
Bangladesh	South-East Asia	Lower middle income	No regulation			0
Barbados	Americas	High income	Yes	Yes		1
Belarus	Europe	Upper middle income	Yes	Yes		1
Belgium	Europe	High income	Yes	Yes		1
Belize	Americas	Upper middle income	Unknown			0
Benin	Africa	Lower middle income	Yes	Yes		1
Bhutan	South-East Asia	Lower middle income	Unknown.			0
Bolivia	Americas	Lower middle income	Yes	Yes		1

Bosnia and Herzegovina	Europe	Upper middle income	Unknown		Yes	1
Botswana	Africa	Upper middle income	Unknown			0
Brazil	Americas	Upper middle income	Yes			1
Brunei Darussalam	Western Pacific	High income	Yes	Yes		1
Bulgaria	Europe	Upper middle income	Yes	Yes		1
Burkina Faso	Africa	Low income	Unknown			0
Burundi	Africa	Low income	Unknown			0
Cabo Verde	Africa	Lower middle income	Yes	Yes		1
Cambodia	Western Pacific	Lower middle income	Yes	Yes		1
Cameroon	Africa	Lower middle income	Yes	Yes		1
Canada	Americas	High income	Yes	Yes		1
Central African Republic	Africa	Low income	Unknown			0
Chad	Africa	Low income	Unknown			0
Chile	Americas	High income	Yes	Yes		1
China	Western Pacific	Upper middle income	Yes	Yes		1
Colombia	Americas	Upper middle income	Yes	Yes		1
Comoros	Africa	Lower middle income	Unknown			0
Congo, Dem. Rep.	Africa	Low income	Unknown			0
Congo, Rep.	Africa	Lower middle income	Yes	Yes		1
Cook Islands	Western Pacific	Not in World bank list	Unknown			0
Costa Rica	Americas	Upper middle income	Yes	Yes		1
Côte d’Ivoire	Africa	Lower middle income	Yes	Yes		1
Croatia	Europe	High income	Yes	Yes		1
Cuba	Americas	Upper middle income	Unknown			0
Cyprus	Europe	High income	Yes	Yes		1
Czechia	Europe	High income	Yes	Yes		1
Denmark	Europe	High income	Yes	Yes		1
Djibouti	Eastern Mediterranean	Lower middle income	Unknown			0

Dominica	Americas	Upper middle income	Unknown			0
Dominican Republic	Americas	Upper middle income	Yes	Yes		1
Ecuador	Americas	Upper middle income	Yes	Yes		1
Egypt, Arab Rep.	Eastern Mediterranean	Lower middle income	Yes	Yes		1
El Salvador	Americas	Upper middle income	Yes	Yes		1
Equatorial Guinea	Africa	Upper middle income	Unknown			0
Eritrea	Africa	Low income	Unknown			0
Estonia	Europe	High income	Yes	Yes		1
Eswatini	Africa	Lower middle income	Unknown			0
Ethiopia	Africa	Low income	Yes	Yes		1
Fiji	Western Pacific	Upper middle income	Yes	Yes		1
Finland	Europe	High income	Yes	Yes		1
France	Europe	High income	Yes	Yes		1
Gabon	Africa	Upper middle income	Unknown			0
Gambia, The	Africa	Low income	Yes	Yes		1
Georgia	Europe	Upper middle income	Yes	Yes		1
Germany	Europe	High income	Yes	Yes		1
Ghana	Africa	Lower middle income	Unknown			0
Greece	Europe	High income	Yes	Yes		1
Grenada	Americas	Upper middle income	Unknown			0
Guatemala	Americas	Upper middle income	Unknown			0
Guinea	Africa	Lower middle income	Unknown			0
Guinea-Bissau	Africa	Low income	Unknown			0
Guyana	Americas	High income	Yes	Yes		1
Haiti	Americas	Lower middle income	Unknown			0
Honduras	Americas	Lower middle income	Yes	Yes		1
Hungary	Europe	High income	Yes	Yes		1
Iceland	Europe	High income	Yes	Yes		1
India	South-East Asia	Lower middle income	Yes	Yes		1
Indonesia	South-East Asia	Upper middle income	Yes		Yes	1

Iran, Islamic Rep.	Eastern Mediterranean	Lower middle income	Yes	Yes		1
Iraq	Eastern Mediterranean	Upper middle income	Yes	Yes		1
Ireland	Europe	High income	Yes	Yes		1
Israel	Europe	High income	Yes	Yes		1
Italy	Europe	High income	Yes	Yes		1
Jamaica	Americas	Upper middle income	Yes	Yes		1
Japan	Western Pacific	High income	Yes			1
Jordan	Eastern Mediterranean	Lower middle income	Yes	Yes		1
Kazakhstan	Europe	Upper middle income	Yes	Yes		1
Kenya	Africa	Lower middle income	Yes	Yes		1
Kiribati	Western Pacific	Lower middle income	Unknown			0
Korea, Dem. People's Rep.	South-East Asia	Low income	Yes	Yes		1
Korea, Rep.	Western Pacific	High income	Yes	Yes		1
Kuwait	Eastern Mediterranean	High income	Yes			1
Kyrgyz Republic	Europe	Lower middle income	Yes	Yes		1
Lao PDR	Western Pacific	Lower middle income	Yes	Yes		1
Latvia	Europe	High income	Yes	Yes		1
Lebanon	Eastern Mediterranean	Lower middle income	Yes	Yes		1
Lesotho	Africa	Lower middle income	Unknown			0
Liberia	Africa	Low income	Unknown			0
Libya	Eastern Mediterranean	Upper middle income	Unknown			0
Lithuania	Europe	High income	Yes	Yes		1
Luxembourg	Europe	High income	Yes	Yes		1
Madagascar	Africa	Low income	Unknown			0
Malawi	Africa	Low income	Unknown			0

Malaysia	Western Pacific	Upper middle income	Yes	Yes		1
Maldives	South-East Asia	Upper middle income	Yes			1
Mali	Africa	Low income	Unknown			0
Malta	Europe	High income	Yes	Yes		1
Marshall Islands	Western Pacific	Upper middle income	Unknown			0
Mauritania	Africa	Lower middle income	Unknown			0
Mauritius	Africa	Upper middle income	Yes	Yes		1
Mexico	Americas	Upper middle income	Yes	Yes		1
Micronesia, Fed. Sts.	Western Pacific	Lower middle income	Unknown			0
Moldova	Europe	Upper middle income	Yes	Yes		1
Monaco	Europe	High income	Yes	Yes		1
Mongolia	Western Pacific	Lower middle income	No regulation			0
Montenegro	Europe	Upper middle income	Yes	Yes		1
Morocco	Eastern Mediterranean	Lower middle income	Unknown			0
Mozambique	Africa	Low income	Unknown			0
Myanmar	South-East Asia	Lower middle income	Unknown			0
Namibia	Africa	Upper middle income	Unknown			0
Nauru	Western Pacific	High income	Unknown			0
Nepal	South-East Asia	Lower middle income	Yes	Yes		1
Netherlands	Europe	High income	Yes	Yes		1
New Zealand	Western Pacific	High income	Yes	Yes		1
Nicaragua	Americas	Lower middle income	Yes	Yes		1
Niger	Africa	Low income	Unknown			0
Nigeria	Africa	Lower middle income	No regulation			0
Niue	Western Pacific	Not in World bank list	Yes	Yes		1
North Macedonia	Europe	Upper middle income	Yes	Yes		1

Norway	Europe	High income	Yes	Yes		1
Oman	Eastern Mediterranean	High income	Yes	Yes		1
Pakistan	Eastern Mediterranean	Lower middle income	No Regulation			0
Palau	Western Pacific	Upper middle income	Yes	Yes		1
Panama	Americas	High income	Yes	Yes		1
Papua New Guinea	Western Pacific	Lower middle income	Yes	Yes		1
Paraguay	Americas	Upper middle income	Yes	Yes		1
Peru	Americas	Upper middle income	Unknown			0
Philippines	Western Pacific	Lower middle income	Yes	Yes		1
Poland	Europe	High income	Yes	Yes		1
Portugal	Europe	High income	Yes	Yes		1
Qatar	Eastern Mediterranean	High income	Yes	Yes		1
Romania	Europe	High income	Yes	Yes		1
Russian Federation	Europe	Upper middle income	Yes	Yes		1
Rwanda	Africa	Low income	Unknown			0
Saint Kitts and Nevis	Americas	Not in World bank list	Unknown			0
Saint Lucia	Americas	Not in World bank list	Yes	Yes		1
Saint Vincent and the Grenadines	Americas	Not in World bank list	Unknown			0
Samoa	Western Pacific	Lower middle income	Yes	Yes		1
San Marino	Europe	High income	Yes	Yes		1
São Tomé and Príncipe	Africa	Lower middle income	Unknown			0
Saudi Arabia	Eastern Mediterranean	High income	Yes	Yes		1
Senegal	Africa	Lower middle income	Yes			1
Serbia	Europe	Upper middle income	Yes	Yes		1
Seychelles	Africa	High income	Yes			1

Sierra Leone	Africa	Low income	Unknown			0
Singapore	Western Pacific	High income	Yes	Yes		1
Slovak Republic	Europe	High income	Yes	Yes		1
Slovenia	Europe	High income	Yes	Yes		1
Solomon Islands	Western Pacific	Lower middle income	Unknown			0
Somalia	Eastern Mediterranean	Low income	Unknown			0
South Africa	Africa	Upper middle income	Yes			1
South Sudan	Africa	Low income	Unknown			0
Spain	Europe	High income	Yes	Yes		1
Sri Lanka	South-East Asia	Lower middle income	Yes	Yes		1
Sudan	Eastern Mediterranean	Low income	No regulation			0
Suriname	Americas	Upper middle income	Yes	Yes		1
Sweden	Europe	High income	Yes	Yes		1
Switzerland	Europe	High income	Yes			1
Syrian Arab Republic	Eastern Mediterranean	Low income	Yes	Yes		1
Tajikistan	Europe	Lower middle income	Yes	Yes		1
Tanzania	Africa	Lower middle income	Unknown			0
Thailand	South-East Asia	Upper middle income	Yes	Yes		1
Timor-Leste	South-East Asia	Lower middle income		Yes	Yes	1
Togo	Africa	Low income	Yes	Yes		1
Tonga	Western Pacific	Upper middle income	Unknown			0
Trinidad and Tobago	Americas	High income	Unknown			0
Tunisia	Eastern Mediterranean	Lower middle income	Unknown			0
Türkiye	Europe	Upper middle income	Yes	Yes		1
Turkmenistan	Europe	Upper middle income	Yes	Yes		1

Tuvalu	Western Pacific	Upper middle income	Yes	Yes		1
Uganda	Africa	Low income	Yes	Yes		1
Ukraine	Europe	Lower middle income	Yes	Yes		1
United Arab Emirates	Eastern Mediterranean	High income	Yes	Yes		1
United Kingdom	Europe	High income	Yes	Yes		1
United States	Americas	High income	Yes	Yes		1
Uruguay	Americas	High income	Yes	Yes		1
Uzbekistan	Europe	Lower middle income	Yes	Yes		1
Vanuatu	Western Pacific	Lower middle income	Yes	Yes	Yes	1
Venezuela, RB	Americas	Upper middle income	Yes	Yes		1
Vietnam	Western Pacific	Lower middle income	No regulations		No	0
Yemen, Rep.	Eastern Mediterranean	Low income	No			0
Zambia	Africa	Lower middle income	Unknown.			0
Zimbabwe	Africa	Lower middle income	Unknown.			0

β**Additional information from other sources:**

Bosnia and Herzegovina: FEDERATION OF BOSNIA: THE LAW ON THE CONTROL AND RESTRICTED USE OF TOBACCO, TOBACCO PRODUCTS, AND OTHER SMOKING PRODUCTS (https://assets.tobaccocontrollaws.org/uploads/legislation/Bosnia%20and%20Herzegovina/Bosnia-and-Herzegovina-2022-TC-Law.pdf)

Indonesia: WHO Indonesia: https://www.who.int/indonesia/news/detail/25-04-2024-toward-tighter-controls--regulating-the-next-generation-of-tobacco-products

Timor-Leste: Timor Leste Tobacco Control Law: CTFK Website (https://www.tobaccocontrollaws.org/legislation/timor-leste/e-cigarettes)

Vanuatu: WHO Western Pacific Region (https://iris.who.int/bitstream/handle/10665/376682/WPR-2024-DHP-001-eng.pdf?sequence=1#:~:text=Sale%20of%20e%2Dcigarettes%20has,%2C%20Palau%2C%20Singapore%20and%20Vanuatu.)

Vietnam: CTFK: https://www.tobaccocontrollaws.org/legislation/viet-nam/e-cigarettes

Yemen: E-cigarettes are not regulated in Yemen. They are classified as electronics or other non-tobacco equipment and are taxed according to these designations.

***https://www.globaltobaccocontrol.org/en/policy-scan/e-cigarettes/countries?country=263**

****https://ggtc.world/library/e-cigarette-ban-regulation-global-status-as-of-october-2023**

*****1=Yes; 0=No**

© 2025 Egbe C.O.
